# Mapping physiology: biophysical mechanisms define scales of climate change impacts

**DOI:** 10.1093/conphys/coz028

**Published:** 2019-08-13

**Authors:** Francis Choi, Tarik Gouhier, Fernando Lima, Gil Rilov, Rui Seabra, Brian Helmuth

**Affiliations:** 1 Marine Science Center, Department of Marine and Environmental Sciences, Northeastern University, 430 Nahant Rd, Nahant, MA, USA; 2 CIBIO, Research Center in Biodiversity and Genetic Resources, University of Porto, Campus de Vairão, Vairão, Portugal; 3 National Institute of Oceanography, Israel Oceanography and Limnology Research Institute, Haifa, Israel

**Keywords:** Biomechanics, ecophysiology, environmental heterogeneity, intertidal zone, microclimate, microhabitat

## Abstract

The rocky intertidal zone is a highly dynamic and thermally variable ecosystem, where the combined influences of solar radiation, air temperature and topography can lead to differences greater than 15°C over the scale of centimetres during aerial exposure at low tide. For most intertidal organisms this small-scale heterogeneity in microclimates can have enormous influences on survival and physiological performance. However, the potential ecological importance of environmental heterogeneity in determining ecological responses to climate change remains poorly understood. We present a novel framework for generating spatially explicit models of microclimate heterogeneity and patterns of thermal physiology among interacting organisms. We used drone photogrammetry to create a topographic map (digital elevation model) at a resolution of 2 × 2 cm from an intertidal site in Massachusetts, which was then fed into to a model of incident solar radiation based on sky view factor and solar position. These data were in turn used to drive a heat budget model that estimated hourly surface temperatures over the course of a year (2017). Body temperature layers were then converted to thermal performance layers for organisms, using thermal performance curves, creating ‘physiological landscapes’ that display spatially and temporally explicit patterns of ‘microrefugia’. Our framework shows how non-linear interactions between these layers lead to predictions about organismal performance and survivorship that are distinct from those made using any individual layer (e.g. topography, temperature) alone. We propose a new metric for quantifying the ‘thermal roughness’ of a site (RqT, the root mean square of spatial deviations in temperature), which can be used to quantify spatial and temporal variability in temperature and performance at the site level. These methods facilitate an exploration of the role of micro-topographic variability in driving organismal vulnerability to environmental change using both spatially explicit and frequency-based approaches.

## Teaser

The topography of an organism’s microhabitat can strongly influence the environmental conditions that it experiences. We present a model that allows mapping of thermal microclimates in rocky intertidal systems, facilitating exploration of the role that these environments may play in driving physiological and ecological responses to climate change.

## Introduction

Ongoing climate change is having clear impacts on the abundance, health and distribution of organisms and subsequently on patterns of biodiversity and ecosystem function (Doney *et al.,* 2012; Bonebrake *et al.,* 2018). In an effort to forecast and potentially mitigate some of the worst impacts of these changes, conservation biologists are increasingly turning to forecasting approaches to predict which populations and species are most vulnerable to accelerating climate change (Dong *et al.,* 2017; Sará *et al.,* 2018; Rilov *et al.*, 2019), where environmental change is occurring most rapidly (Sunday *et al.,* 2015; Brito-Morales *et al.,* 2018) and what measures might be enacted to protect threatened species and ecosystems by either reducing the effects of non-climatic stressors such as development and overharvesting (Przeslawski *et al.,* 2005; Sará *et al.,* 2018) or by prioritizing the protection of refugia (Morelli *et al.,* 2017). Laboratory- and field-based physiological methods are playing an increasingly important role in our understanding of how climate change is affecting natural and managed systems and of the range of possible conservation interventions that can be enacted (Seebacher and Franklin, 2012; Chown and Gaston, 2016; Marn *et al.,* 2017; Teal *et al.,* 2018; Rilov *et al.,* 2019).

At the same time, our ability to quantify, model and forecast the physical parameters, such as temperature, nutrients, rainfall and ocean pH, that drive these observed and projected changes continues to improve (Shukla, 1998; Maclean *et al.*, 2019). For example, the thermal limits of key species can be measured under natural and controlled temperatures and then compared against contemporary and projected patterns of ‘environmental temperature’ to estimate ‘thermal safety margins’, the difference between what an organism experiences in the field relative to its tolerance (Kingsolver, 2009; Polgar *et al.,* 2015; Bruno *et al.,* 2018). Yet there remain frequent mismatches between what are often very careful and detailed measurements of physiological vulnerability and the scale at which environmental measurements and projections are made in the field (e.g. see discussions in Sears *et al.* 2011; Boyd *et al.,* 2016; Torossian *et al.,* 2016; Garcia *et al.,* 2019). For example, projections of vulnerability based on climatic (or even annual) means have little hope of forecasting the effects of much higher frequency variability in environmental conditions such as heat waves and cold snaps (Wethey *et al.,* 2011a; Roitberg and Mangel, 2016), which are themselves becoming more frequent under anthropogenic climate change. Comparably, remote sensing is frequently unable to capture environmental conditions at the level of microhabitats (Sears *et al.,* 2011; Faye *et al.,* 2016; Maclean *et al.*, 2019) that ultimately drive biotic responses (Scheffers *et al.,* 2014a; Storlie *et al.,* 2014).

This study explores this fundamental disconnect (Denny and Helmuth, 2009; Helmuth *et al.,* 2010) and describes how an understanding of the mechanisms by which plants and animals interact with their physical environment (i.e. ecophysiology) can lend insight into how these scales can be better aligned (Helmuth *et al.,* 2005; Flynn *et al.,* 2012; Harley, 2013; Scheffers *et al.,* 2014b; Jurgens and Gaylord, 2017). Specifically, we examine how biophysical approaches (photogrammetry coupled with heat budget modelling and physiological measurements) can be used to address questions of scale (Sears *et al.,* 2011), both in terms of how we measure the physical environment (Potter *et al.,* 2013; Maclean *et al.*, 2019) and of how we measure physiological, behavioural and ecological responses to environmental conditions (Flynn *et al.,* 2012; Woods *et al.,* 2015; Rebaudo *et al.,* 2016).

### Scale, environmental heterogeneity and physiological response

Questions about scale and especially about the importance of environmental heterogeneity (EH) have a rich history in ecology spanning many decades (Schneider, 2001). Relationships between EH in space and time and biological parameters such as distribution, abundance, biomass, biodiversity and resilience in the face of perturbations have been explored in habitats as diverse as coral reefs (Keith *et al.,* 2013), open ocean systems (Boyd *et al.,* 2016), terrestrial forests (van Rensburg *et al.,* 2002; Morelli *et al.,* 2018), deserts (Migliore *et al.,* 2013), deep sea benthos (Williams *et al.,* 2010), seamounts (Clark *et al.,* 2012) and rocky reefs (Matias *et al.,* 2011). Understanding the role of EH in these processes has taken on new significance in this era of ongoing rapid environmental change (Boyd *et al.,* 2016; Kelly, 2019), and a number of studies have begun to explore the role that microclimates may play in mediating larger-scale climatic drivers (Sears *et al.* 2011, 2016; Potter *et al.,* 2013; Hannah *et al.,* 2014; Maclean *et al.,* 2015). For example, sites with high levels of EH (and thus presumably species richness) may represent priority areas for conservation or similarly serve as ‘rescue sites’ following extreme events (Hanski and Ovaskainen, 2000; Roberts *et al.,* 2017). EH driven by structuring species such as bivalves and macroalgae has been shown to override large-scale geographic trends in environmental conditions (Jurgens and Gaylord, 2017), with implications for predictions of range shifts and resilience to climate change. Increasing surface complexity has also been explored as a mechanism to increase biodiversity on seawalls (Chapman and Bulleri, 2003; Loke *et al.,* 2017).

Methods for measuring structural complexity have also expanded rapidly in recent years with the easy access of small unmanned aerial systems (sUAS), i.e. ‘drones’. sUASs are now frequently used to construct digital elevation models (DEMs) and other types of 3D virtual models, providing new tools for exploring different ecological descriptors of various ecosystems. These include structural complexity in coral reefs (Gonzalez-Rivero *et al.,* 2017), habitat categorization in rocky shores (Garza, 2016) and in freshwater fish habitats (Kalacska *et al.,* 2018), species distributions and biodiversity surveys in forest ecosystems (Torossian *et al.,* 2016) and thermal distribution in agricultural landscapes (Faye *et al.,* 2016).

An increasing number of physiological studies are similarly beginning to consider the scales over which physiological responses can vary in time and space (Dowd *et al.,* 2013; Malishev *et al.,* 2017). Dong *et al.* (2017), for example, measured cardiac function in three species of intertidal snails and showed that intraspecific variation (physiological polymorphism) in flat-line temperatures exceeded interspecific differences. They also showed that, congruently, differences in habitat temperatures within sites far exceeded differences among sites along a 12° gradient in latitude on the coast of China. Denny *et al.* (2011) reported differences in within-site microhabitat temperatures that exceeded those reported over 14° of latitude on the west coast of North America, and Seabra *et al.* (2011) found similar differences on the Iberian coast. These combinations of heterogeneity in environmental conditions (microclimates) with inter-individual variability in physiological sensitivity have significant implications for how we envision selective regimes (Schmidt *et al.,* 2000; Lawson *et al.,* 2014; Denny, 2018; Kelly, 2019), notably in ways that cannot be predicted when physiological tolerance is considered as a species trait with no inter-individual variability (e.g. Diamond *et al.,* 2012) or when an environmental parameter such as temperature is measured in one specific location and then used to represent an entire site (e.g. Enquist *et al.,* 2017).

Despite the large number of studies exploring the role of microhabitats and EH, comparatively few have quantitatively measured how structural complexity actually mediates the local microenvironments (microclimates) of plants and animals (but see e.g. Sears *et al.,* 2011; Barton and Terblanche, 2014; Kearney *et al.,* 2014; Hayford *et al.,* 2015; Pincebourde *et al.,* 2016; Jurgens and Gaylord, 2017; Maclean *et al.*, 2019). A recent meta-analysis by Ortega *et al.* (2018) showed that a majority of studies over the last two decades exploring EH/structural complexity reported a positive relationship between EH and species richness (S). However, their analysis also showed that, somewhat surprisingly, very few experiments have quantitatively explored the underlying mechanisms driving the EH and S relationship, leading them to the conclusion that this area of research was still in its ‘infancy’. Other reviews have come to similar conclusions, again pointing to a dearth of studies focused on mechanism (Kovalenko *et al.,* 2012; Loke *et al.,* 2015). This paucity of research serves as a major impediment to our understanding of how microenvironments affect community resilience to climate change (Potter *et al.,* 2013; Woods *et al.,* 2015).

To a large extent, these gaps in our understanding exist for the simple reason that modelling and measuring environmental conditions at scales that are both relevant to organismal physiology (i.e. microclimates), but that can also be applied over scales sufficient to detect biogeographic shifts in response to environmental change, are usually impractical (Bates *et al.,* 2018). Fixed sensors can record time series at a single location with high accuracy but may not be reflective of the actual spatial diversity in environmental conditions (Denny *et al.,* 2011; Miller and Dowd, 2017). Infrared cameras can survey large areas (e.g. with drones) but typically can only record environmental conditions at a single point in time, unless they are mounted in place for extended periods (Scherrer and Koerner, 2010). Satellites can accomplish both feats to limited degrees but with spatial (e.g. 1-100 km^2^) and temporal (e.g. 6 hr) resolutions that may be irrelevant to the organism in question (Potter *et al.,* 2013; Simó *et al.,* 2016; Geller *et al.,* 2017).

### A case study: rocky intertidal zones

Perhaps nowhere are the challenges inherent in measuring microclimates as apparent as in rocky intertidal zones, areas with enormous spatial and temporal variation in factors such as wave force (Helmuth and Denny, 2003), oxygen (Frieder *et al.,* 2012), pH (Hofmann *et al.,* 2011; Baumann and Smith, 2018) and, especially, temperature during aerial exposure at low tide (Denny *et al.,* 2011). Body temperature is one of the most universal determinants of a plant or animal’s physiological performance and survival (Somero, 2002, 2010; Sinclair *et al.,* 2016), and the rocky intertidal zone has long served as a model ecosystem for exploring the relationship between temperature and ecological responses over local and geographic scales (Connell, 1972; Sorte *et al.,* 2019). A number of recent studies have documented that many species of intertidal invertebrates—animals whose ancestors evolved in a fully aquatic environment—currently live very close to their thermal limits (Somero, 2002; Wethey and Woodin, 2008; Mislan *et al.,* 2014). Large-scale mortality events in response to elevated low-tide temperatures have been reported (e.g. Harley, 2008), in some cases on a recurring basis (Williams and Morritt, 1995; Firth and Williams, 2009).

Notably, it is the temperature of an organism’s body that ultimately drives physiological responses and not the temperature of its surrounding environment per se. Kearney (2006) defines this as the ‘niche’ temperature, as differentiated from aspects of the ‘environment’ such as local air temperature. This distinction is not trivial, as the body temperature of ectothermic plants and animals can be very different from local air temperature, especially when exposed to direct sunlight (Fitzhenry *et al.,* 2004; Chapperon *et al.,* 2016). In air at low tide, body temperatures are driven by multiple environmental factors including solar radiation, air temperature, humidity, wind speed and cloud cover (Denny *et al.,* 2006) and are affected by the characteristics of the organism such as colour, shape, mass and material properties (Helmuth, 2002). For organisms with large areas of their body adhered to the substratum (limpets, barnacles, some snails and small mussels) body temperature usually closely tracks the temperature of the rock surface (Wethey, 2002), which on sunny days is much hotter than the air above it. For larger animals (e.g. large bivalves) or organisms with a wetted surface (e.g. seastars) body temperatures can be substantially different from either rock or air (Broitman *et al.,* 2009; Miller and Dowd, 2017).

In terrestrial and intertidal systems, by far the most significant driver of ectotherm temperature is exposure to direct solar radiation (Helmuth, 1998; Scheffers *et al.,* 2017; Maclean *et al.*, 2019). While mortality events are often associated with episodes of high air temperature (Mislan *et al.,* 2014, Sorte *et al.,* 2019) these only occur when both air temperature is elevated and solar radiation is at a maximum; both conditions are typically required in order for large-scale mortality to occur, and thus elevated air temperature alone is an effective indicator of mortality events *only* when it occurs on cloud free days with maximum solar radiation (Gilman *et al.,* 2006, Mislan *et al.,* 2014). Because of the importance of solar radiation (Marshall *et al.,* 2010; Chapperon *et al.,* 2016), the difference in temperature between an animal on a poleward-facing (shaded) microsite and one on a nearby equatorial-facing (sunny) microsite can easily exceed 15°C (Helmuth and Hofmann, 2001; Sears *et al.,* 2011). Such large differences in thermal environments among shaded and unshaded surfaces have been shown to lead to even larger differences in physiology (Jimenez *et al*., 2015; Miller *et al.,* 2015), survival (Harley, 2008), abundance (Meager *et al*., 2011) and selection for different species and genotypes (Schmidt *et al*., 2000; Schneider and Helmuth, 2007; Seabra *et al*., 2011). This non-linear translation between small changes in body temperature and large changes in physiological performance is best conceptualized using Jensen’s inequality (Martin and Huey, 2008; Denny, 2019), which further highlights the dangers of using temporal or spatial averages to predict biological responses. Over geographic scales, the presence of shaded refugia has been proposed as a mechanism that allows species to extend their range boundaries beyond what they could survive if only sunny habitats were available (Sunday *et al.,* 2014, Lima *et al.,* 2016).

Aerial temperatures at low tide in intertidal zones can often be difficult to measure. Thermocouple sensors (often used in terrestrial studies) are easily broken by wave action, and drones equipped with infrared cameras can take snapshot measurements of intertidal rock and organism temperatures but only for limited amounts of time (Lathlean *et al.,* 2012, Seuront *et al.,* 2018). Biomimic sensors have provided significant insights into the temperature that intertidal invertebrates experience (reviewed in Judge*,* 2018), but measurements are typically restricted by the number of sensors that can be deployed at any given site (Helmuth *et al.,* 2016) or by the duration of deployment (Denny *et al.,* 2011). However, when combined with heat budget models that use environmental inputs from weather station data or gridded (reanalysed) meteorological data (Mislan and Wethey, 2011; Dong *et al.,* 2017), intertidal animal temperatures can be estimated over a range of scales. Several mathematical (heat budget) models are now available to convert weather data into estimates of intertidal organism temperature (Bell, 1995; Helmuth, 1998, 1999; Denny and Harley, 2006; Szathmary *et al.,* 2009; Helmuth *et al.,* 2011; Iacarella and Helmuth, 2011; Sarà *et al.,* 2011; Wethey *et al.,* 2011b; Mislan and Wethey, 2015; Marshall *et al.,* 2015; Kish *et al.,* 2016, Dong *et al.,* 2017). These models range in complexity from simple regression-based approaches (Elvin and Gonor, 1979; Kish *et al.,* 2016) to much more sophisticated land surface–based models (Wethey *et al.,* 2011b; Mislan and Wethey, 2015).

**Figure 1:**
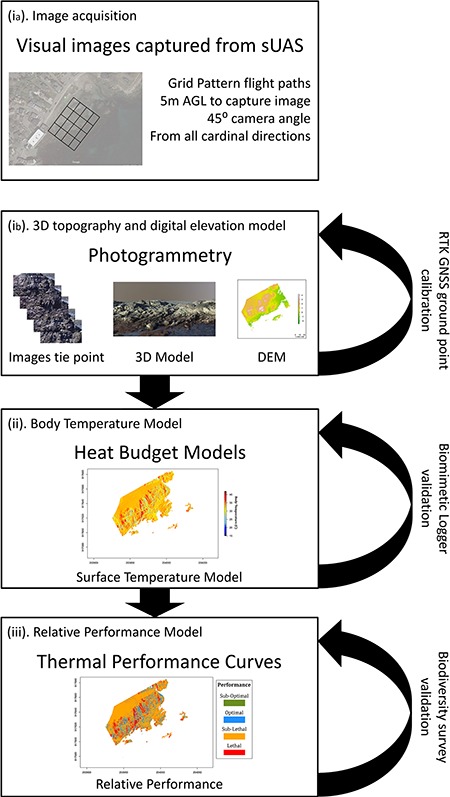
Systematic workflow of the integrative framework to analyse thermal landscapes. Following the framework steps: (**i**) create DEM through (**ia**) image acquisition with sUAS and (**ib**) image processing through photogrammetry. (**ii**) Estimate surface temperatures with heat budget model. Finally, (**iii**) estimate relative performance with thermal performance curve.

**Figure 2:**
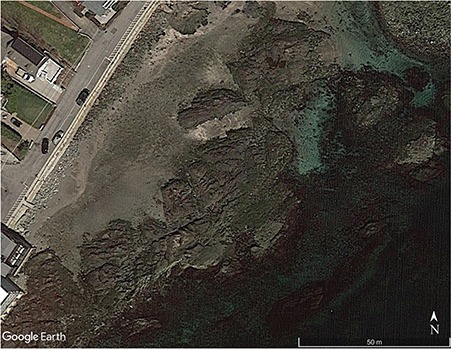
Fort Beach in Marblehead, MA USA (42.508° N, 70.843° W).

To date, however, most heat budget models (but see Sears *et al.,* 2011; Kearney *et al.,* 2014; Dong *et al.,* 2017; Kearney and Porter, 2017; Maclean *et al.,* 2019) have tended to ignore the role of small-scale microclimates and especially the role of incident solar radiation in driving within-site variation in microhabitat temperatures. Here we present an integrative framework that utilizes DEMs and heat budget models to quantify microhabitat temperatures (Fig. 1), using a ~ 50 m × 100 m intertidal site on the Northeast coast of the US (Fort Beach in Marblehead, MA; 42.508° N, 70.843° W; Fig. 2) as a case study. Using this approach we characterize the full suite of microenvironments at this rocky intertidal site as a function of structural complexity and discuss how this approach can be used to explore the role of microclimate in driving patterns of behaviour, physiological performance and mortality. This framework includes (i) capturing fine-scale topographic data using drone photography to produce 3D models and DEMs, (ii) transforming large-scale weather data to account for surface orientation to solar radiation, (iii) identifying microhabitat temperatures using a heat budget model and (iv) comparing predictions of body temperature against thermal performance models to make spatially and temporally explicit predictions of relative physiological performance.

## Spatial model framework

### DEMs

**Figure 3:**
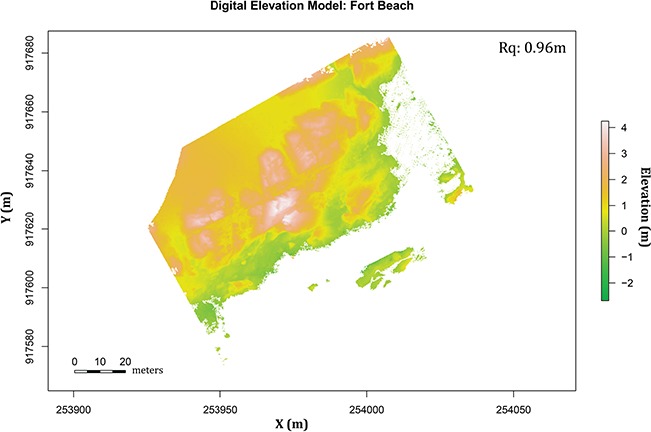
DEM of Fort Beach. Colours represent elevation across the model, with max elevation at 4.3 m above mean lower low water. Rq, the surface roughness, is calculated to be 0.96 m at Fort Beach. Note that blank spaces represent coverage by water at low tide.

In the past, fine-scale topographic data have been difficult and expensive to obtain, and DEMs were produced using methods such as real-time kinetic (RTK) Global Positioning System (GPS) mapping (Morton *et al.,* 1999, Freeman *et al.,* 2004), Light Detection and Ranging (LiDAR) (Polat and Uysal, 2015) and radar interferometry or photogrammetry methods (Colomina *et al.,* 2008; Eisenbeiss, 2009). The advancement and accessibility of sUAS have allowed some of these methods, especially photogrammetry and LiDAR, to become more accessible to field biologists (Faye *et al.,* 2016). In this framework, fine-scale topographic data are mapped using an sUAS flown at low elevations (e.g. 5 m) above ground level, in a grid format and from various directions, to capture numerous high-resolution images from a camera angle of 45°. We used this approach to capture a total of 528 photos at Fort Beach. This high number of images is needed to capture all the topographic characteristics and to allow photogrammetric methods to estimate aspects, slopes and height of these topographies at a resolution of 2 × 2 cm. Agisoft Photoscan Pro photogrammetry software was used to convert footage from the sUAS to 3D virtual models, which were exported as DEMs (Fig. 3). A detailed workflow of photogrammetric methods, such as matching image tie points and generating dense point clouds, can be found in James and Robson (2012) and Faye *et al.* (2016). To provide reference points for the photogrammetry software, multiple precise (sub-centimetre accuracy) GPS coordinates, including elevation, of topographic features were recorded using a Trimble RTK global navigation satellite system. Figure 3 shows a DEM produced through photogrammetry of the Fort Beach site.

### Surface temperature model

**Figure 4:**
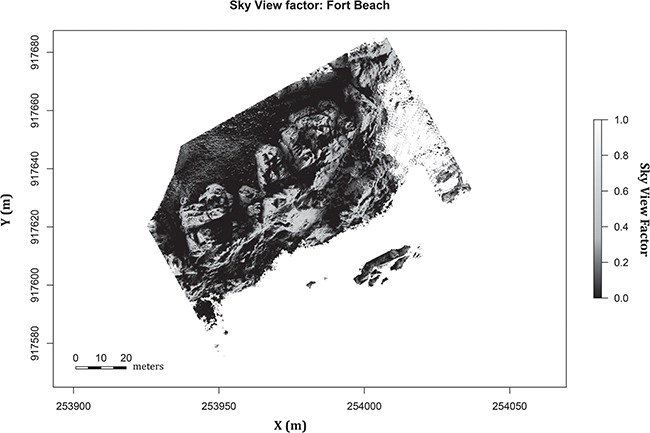
SVF of Fort Beach. SVF is calculated by using 32 search vectors per pixel and a maximum search radius of 5 m per vector. SVF ranges from 0 to 1 for each pixel of the map, 0 being 100% shaded and 1 being 100% exposed to the sky. Note that blank spaces represent coverage by water at low tide.

To date, most heat budget models for ecological forecasting have failed to incorporate complex topography on a spatially explicit basis (but see Sears *et al.*, 2011, who used complex simulated surfaces, and Maclean *et al.,* 2019, who calculated topographic complexity at scales of ~ 10 m). The major advancement in the framework presented here is to use DEMs from actual sites to estimate direct and reflected solar radiation on each surface element at very fine spatial scales, which can then be used as inputs to a heat budget model. This approach is accomplished by downscaling total solar radiation measurements recorded from a local weather station or from (reanalysed) gridded meteorological data (Mislan and Wethey, 2011; Dong *et al.,* 2017; Maclean *et al.,* 2019) to estimate spatial and temporal distribution of solar radiation at a local level. Downscaling is a multi-step process that involves (i) generating solar geometry: plotting the locations of the sun relative to each pixel throughout the day (via latitude, azimuth and motion of the sun) and adjusting the intensity of direct solar radiation through calculations of the solar azimuth and solar zenith (Braun and Mitchell*,* 1983); (ii) calculating albedo effects and the atmosphere diffusion factor (Bindi *et al.,* 1992) to describe the effect of direct solar radiation entering through the atmosphere and hitting the surface; and (iii) measuring and incorporating DEM topographic variables [elevation, slope, aspect and sky view factor (SVF)] that define the distribution of solar radiation on complex topography (Tovar-Pescador *et al.,* 2006). The most complex of these parameters, the SVF, is widely used to measure shading in urban environments, by hillsides, and in forest canopies (Holmer *et al.,* 2001; Zakšek *et al.,* 2011; Polo López *et al.,* 2016; Hoylman *et al.,* 2018), but it is relatively under-utilized in heat budget modelling and ecological forecasting. Calculated by using vectors and their distances on a hemisphere per pixel, SVF represents the amount of sky each pixel is exposed to, an index from 0 to 1, where 0 is completely shaded and 1 is completely exposed to the sky (Fig. 4). Hence, this metric helps create a shade/exposed solar radiation relationship between each pixel and its neighbors (Zhang *et al.,* 2017). Incorporating SVF local shadowing effects into downscaling can provide a more robust model for solar radiation on complex topography (Matzarakis *et al.,* 2007; Zakšek *et al.,* 2011; Polo López *et al.,* 2016).

**Figure 5:**
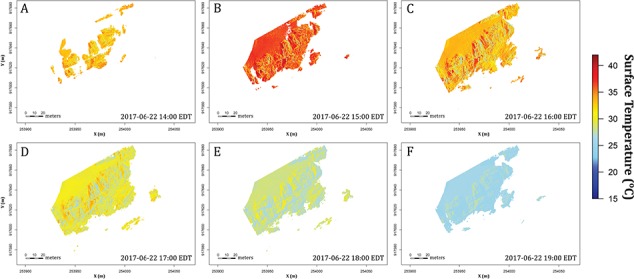
Surface temperature models at Fort Beach. Figure presents the low-tide period during the hottest day of 2017 (22 June) from (**A**) 14:00 EDT to (**F**) 19:00 EDT (solar noon at this location is at 13:00 EDT). Surface temperature distribution is highly variable through space and time. Note that blank spaces represent coverage by water at high tide.

We downscaled solar radiation data obtained from the Climate Forecast System Reanalysis (CFSR) meteorological database (Mislan and Wethey, 2011). These downscaled data were used as inputs with other weather data from CFSR (air temperature, wind speed and water temperature) to a heat budget model (described in Dong *et al.,* 2017) to estimate surface temperature distribution spatially (sub-centimetre) and temporally (hourly) across all the microhabitats within the site. Still tidal elevation predictions (XTide; http://www.flaterco.com/xtide/) were used to estimate when microsites were submerged and when they were aerially exposed. This modified heat flux model (Helmuth, 1998; Dong *et al.,* 2017) was used to estimate surface temperatures across the entire rocky shore of Fort Beach (Fig. 5). We ran hourly simulations for an entire year (2017, shown in Supplement A) but here focus on the 2 days of the year with the highest surface temperature estimations, 22 June and 23 June. While these predictions were not fully validated—an exercise that would require high-resolution sampling over time using sensors and/or repeated images using infrared thermography—the results are consistent with the range of temperatures reported in past studies for other sites (Denny *et al.,* 2011; Seabra *et al.,* 2011; Dong *et al.,* 2017). During the hottest surface day in 2017 at Fort Beach (22 June), where the peak global solar radiation reached 883 Wm^−2^, modeled surface temperatures ranged from 25.7°C to 41.4°C, consistent with the temperature range measured with *in situ* loggers from this site in sunny and shaded microhabitats (unpublished data). Notably, maximum air temperature on this day was only 27.8°C, pointing to the importance of solar radiation. Conversely, the highest air temperature of the year occurred on 12 June (34.7°C), but surface temperature at Fort Beach remained low because most of the beach was submerged during peak solar periods (Helmuth *et al.,* 2002).

It should be noted that we used the model to estimate only rock surface temperature, which has been used as a direct proxy for the body temperature of animals with a high proportion of their body in contact with the rock, for example limpets (Seabra *et al.,* 2011) and barnacles (Wethey, 2002). For some animals, especially larger animals such as mussels (Helmuth, 1998) and seastars (Szathmary *et al.,* 2009), rock temperature is not equivalent to animal temperature, but the same approach used here can be applied on an organism-specific level by modifying model parameters (e.g. Szathmary *et al.,* 2009; Dong *et al.,* 2017). For simplicity we also assume that animals are sessile during low tide, a reasonable assumption for many organisms that ‘hunker in place’ during aerial exposure, but less realistic for other, more mobile organisms (Williams and Morritt, 1995; Monaco *et al.,* 2015). Notably, the model presented here does not include the role of water retention in tidepools or other small features of the rock surface, which could provide additional refugia through cooling from evaporation of saltwater, albeit at the potential cost of physiological stress from high salinity.

### Relative performance model

**Figure 6:**
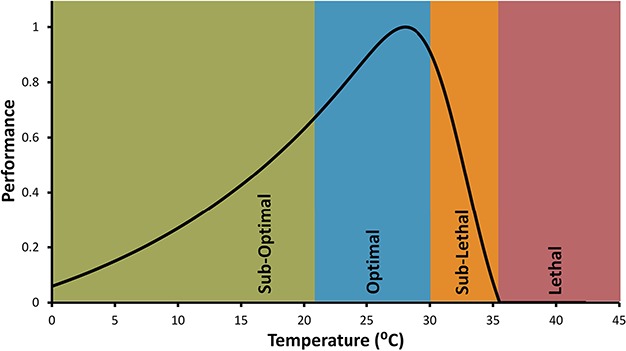
A typical TPC for intertidal organisms. TPC is divided into different performance categories to identify the levels of thermal stress. This TPC has its sub-optimal range from 0°C to 21.2°C; optimal range is from 21.2°C to 30.1°C with optimal temperature (T_opt_) at 28°C; sub-lethal range from 30.1°C to 35°C and finally high lethal temperature is >35°C.

The ultimate goal of the approach described here is to provide a method of mapping physiological performance and survival over space and time using DEMs. The simplest approach to doing so is to convert body temperature to some metric of relative performance using a thermal performance curve (TPC), which in turn is based on indirect metrics of fitness such as respiration, movement or heart rate (Huey and Slatkin, 1976; Huey and Stevenson, 1979; Sinclair *et al.,* 2016). TPCs describe the non-linear relationship (Martin and Huey, 2008; Denny, 2017) between temperature and these physiological rates, most typically as a curve in which performance rises slowly with temperature up to an optimal level, T_opt_, and then drops rapidly (Angilletta, 2006, 2009; Kish *et al.,* 2016) (Fig. 6). The assumption that TPCs remain constant over time (i.e. no capacity for acclimatization) or that they can be based on a single performance metric such as heart beat rate or behaviour is problematic and has been discussed by several authors (Kingsolver and Woods, 2016; Sinclair *et al.,* 2016; Stoffels *et al.,* 2016; Monaco *et al.,* 2017). However, they provide an easy first-cut approach in estimating performance based on body temperature. More sophisticated approaches could, for example, alter the spatial distribution of individual TPCs of animals based on their thermal history (Kingsolver and Woods, 2016) or could use physiological approaches such as Dynamic Energy Budget models that explicitly account for thermal history (Augustine and Kooijman, 2019).

**Figure 7:**
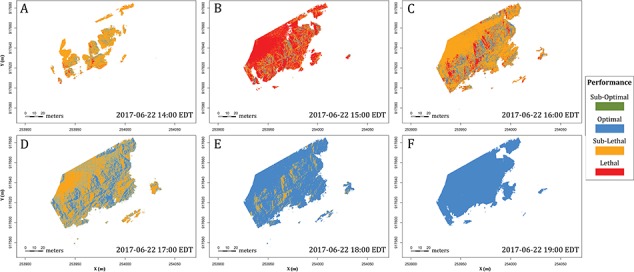
Relative performance models at Fort Beach. The figure describes variation in performance distribution spatially as well as throughout a low-tide period. Performances are categorized into sections for sub-optimal (green), optimal (blue), sub-lethal (orange) and lethal (red). Colours correspond to the categories in Fig. 6, and blank spaces correspond to water at high tide.

TPCs for intertidal species can be estimated through physiological experiments measuring movement speed (Tepler *et al.,* 2011), respiration rate (Marshall *et al.,* 2010) and heart rate (Dong *et al.,* 2017), and there is a growing body of data for various intertidal species. Figure 7 is an example of a spatially explicit relative performance model that combines a generic TPC with a T_opt_ of 28°C and a lethal temperature of 35°C (Fig. 6), with a DEM from the Fort Beach site over the course of 6 h. Here, a performance is divided into categories of suboptimal, optimal, sublethal and lethal (after Kish *et al.,* 2016).

## Applications of the approach

**Figure 8:**
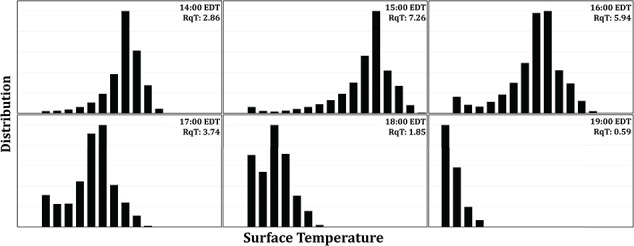
Frequency distribution of surface temperature across the six timestamps on 22 June 2017 shown in Fig. 5. Surface temperature distribution range from 22°C to 41°C (from left to right at each panel). Panels describe variations in the peaks and shape at each timestamp. The patterns of surface temperature distribution can be quantify through thermal roughness (RqT), where the higher RqT means higher distribution of temperatures across the site.

**Figure 9:**
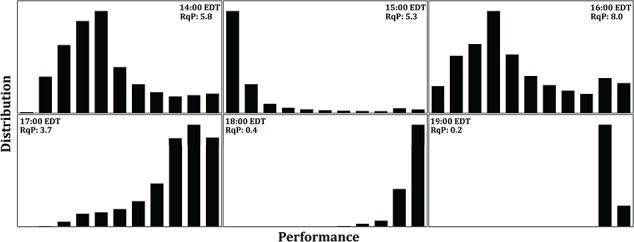
Frequency distribution of relative performance across the six timestamps on 22 June 2017 shown in Fig. 7. Relative performance distribution range from 0 to 1 (from left to right at each panel). Panels describe variations in the peaks and shape at each timestamp. The patterns of performance distribution can be quantify through performance roughness (RqP), where the higher RqP means higher distribution of performances across the site.

The model layers presented here—structural (microhabitat), thermal (microclimate) and physiological—provide an example of the non-linear way in which EH affects the performance and survival of organisms (Martin and Huey, 2008; Denny, 2017) and argues that knowledge of one metric (e.g. structure) does not necessarily provide a full understanding of the mechanisms by which microhabitats drive thermal ecology. As described by Stein and Kreft (2014) it has been commonly assumed that habitat (structural) complexity can serve as a direct proxy for EH and microclimate diversity (but see Angilletta, 2009; Armstrong *et al.,* 2013; Scheffers *et al.,* 2017 for counter examples). Certainly, physical structures such as forests, coral reefs, bivalve beds and algal canopies can drive EH through their influence on processes such as shading (Reed and Foster, 1984) and reductions in wind or water flow (Guichard *et al.,* 2001, Lenihan *et al.,* 2008; Gaylord et al., 2012), but these relationships can be far more complex and non-linear than is generally appreciated (Layton *et al.*, 2019). All of this points to the issue that the many approaches that have been adopted for quantifying habitat structural complexity (Frost *et al.,* 2005; Dibble and Thomaz, 2009), while likely correlated to varying degrees with microclimate diversity (Ehbrecht *et al.,* 2017), cannot be assumed to serve as a direct proxy for environmental conditions at the level of the organism (Meager and Schlacher, 2013; Stein and Kreft, 2014). In other words, the knowledge of structural complexity alone does not automatically translate into an understanding of the heterogeneity of environmental conditions that ultimately drive physiological performance, survival and biodiversity nor does it account for the non-linear relationship between body temperature and physiological performance, i.e. Jensen’s inequality (Martin and Huey, 2008; Denny, 2017). These issues are highlighted in Figs 8 and 9, which show frequency distributions of surface temperature (Fig. 8) and relative performance (Fig. 9) over the course of a 6-hour low-tide exposure. Such frequency distributions provide considerably more information than simply bracketing the range of temperatures using the coolest (full shade) and hottest (full sun) microsites, an approach that has been used in previous studies (e.g. Marshall *et al.,* 2015, Dong *et al.,* 2017).

**Figure 10:**
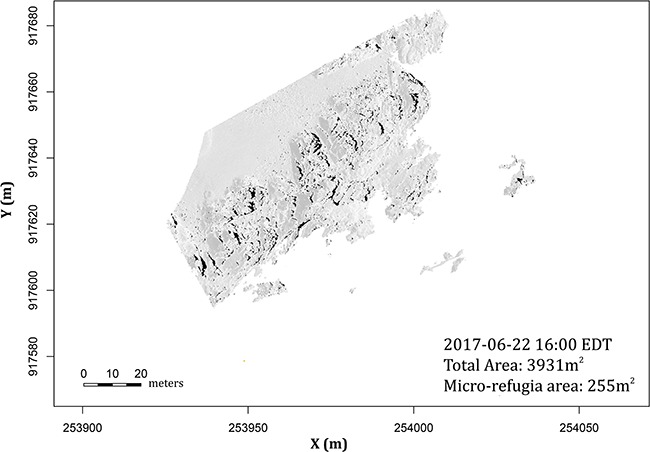
Surface temperature distribution on 22 June 2017 at 16:00 EDT. This figure compares the total area (greyscale) during low tide to micro-refugia area shown in black (in this scenario, micro-refugia are defined as sites where surface temperature < 28°C). This figure shows how spatial models can reveal the scarcity of micro-refugia at the rocky shores during a heat event.

As a first attempt at rectifying the potential disconnect between the different data layers (structural, thermal and physiological) we calculated a common metric of surface roughness, Rq, the root mean square (RMS) of deviations in surface elevation above the mean plane (Thomas, 1999). For the Fort Beach site, the estimate for Rq was calculated as 0.96 (Fig. 3). Here we propose an analogous metric of ‘thermal roughness’ (RqT) that comparably calculates the RMS of deviations in temperature within a site (Fig. 8). These will change over time, ranging from very low values (Fig. 8A) when the sun is low, or immediately after emersion, to peak values when thermal heterogeneity at the site is the highest (Fig. 8). In the example shown in Fig. 8, RqT ranges from 0.59 to 7.26 over the course of 6 h. Similar approaches can be used to estimate performance variability (RqP; Fig. 9). Both of these approaches could be used to quantify, for example, variability in selective regimes at a site over the course of a day, season, year or over longer climatic scales.

Spatially explicit body temperature and physiological performance modelling can further be used to quantify areas of micro-refugia (Monaco *et al.,* 2015) by applying a series of threshold temperatures. For example, using a definition of ‘refugia’ as any temperature under 28°C, the area calculated from the Fort Beach site at 16:00 Eastern Daylight Time (EDT) (20:00 Greenwich Mean Time (GMT)) is 255 m^2^, less than 7% of the total aerially exposed site area (3931 m^2^; Fig. 10). Simulations of relative performance provide the opportunity for an even more in-depth examination of the ecological consequences of temperature variability, by identifying areas of lethal, sub-optimal temperature, optimal temperature, sub-lethal temperature and lethal temperature (Fig. 7).

**Figure 11:**
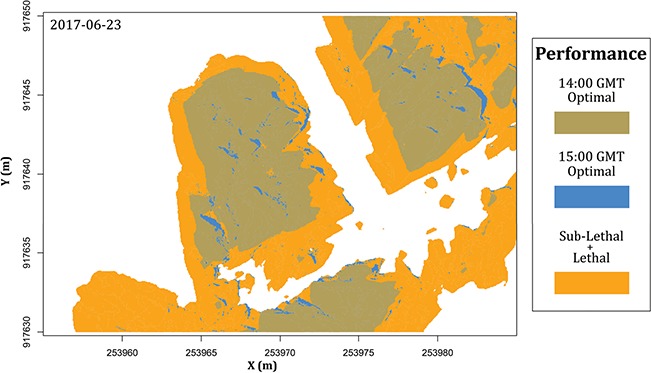
Changes in optimal performance areas within 1 h on 23 June 2017. Blue–brown represents optimal performance areas at 14:00 EDT, while blue represents optimal performance areas at 15:00 EDT. Orange represents areas of sub-lethal or lethal performance. Relative performance is based on an intertidal organism TPC with a T_opt_ of 28°C. Temporal comparisons of relative performance can be an opportunity to describe thermal behaviour for mobile species. The difference of optimal performance area between timestamps represents potential thermal corridors for mobile species.

**Figure 12:**
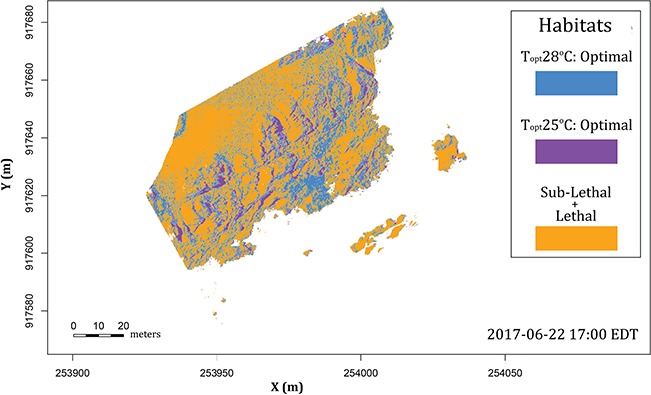
Habitat structure based on the relative performance of interacting species. Blue represents a prey species with a TPC of 28°C; purple represents a predator or dominant species with a TPC of 25°C. Species blue is bounded by not only the habitat of the predator but also its own upper thermal limits, shown in orange.

While these calculations ignore the role that body size can play in defining micro-refugia (Meager and Schlacher, 2013), this aspect could be included by considering only sites larger than a minimum area related to body size or, conversely, by spatially averaging surface temperatures to account for large organisms that themselves shade the substrate (such as seastars; Monaco *et al.,* 2015). This approach also offers the opportunity to explore the role of behavioural thermoregulation (Williams *et al.,* 2005; Sunday *et al.*, 2014), for example by identifying thermally protected ‘corridors’ and ‘barriers’ in the landscape (Fig. 11). For highly mobile species, movement to protected microhabitats during extreme conditions at low tide can serve as an effective form of behavioural thermoregulation (Iacarella and Helmuth, 2011; Darnell *et al.,* 2015; Sears *et al.,* 2016). For other, more slow-moving species, the decision of whether or not to shelter in a shaded microhabitat occurs during the preceding high tide so that at low tide they may move very little or not at all. For example, many snail species pre-emptively move to crevices and other shaded areas during high tide to avoid extreme temperatures during the following low tide (Marshall *et al.,* 2010; Cartwright and Williams, 2012; Ng *et al.,* 2017). Corridors for pre-emptive movement based on thermoregulation have been studied in the field using infrared cameras (Chapperon and Seuront, 2011), with radio-frequency identification tags (Hayford *et al.,* 2015, 2018) and survey classification (Monaco *et al.,* 2015).

The approach shown here also allows an opportunity to explore the potential for competition for refugia sites among interacting organisms (as has been done much more extensively for lizards and snakes, e.g. Goller *et al.,* 2014; Sears *et al.,* 2016; Lopez-Alcaide *et al.,* 2017). For example, Fig. 12 shows the location of refugia available to an organism—for example a predator or dominant competitor—with a lower thermal optimum (here, T_opt_ = 25°C) and lethal limit, vs. one by an organism with a higher thermal limit (T_opt_ = 28°C), its prey. In this example, as in Fig. 12, the predator/dominant competitor dominates in cooler microhabitats, and the prey/subordinate competitor can only persist in microrefugia that are unfavourable to the predator/competitive dominant (Wethey, 1983, 1984). Using a spatially explicit model for multiple species can be useful to predict patterns of vertical zonation that results from the interaction between the physical environment experienced by an organism and its physiological limits and biotic interactions (Lewis, 1964; Connell, 1972; Wethey, 1983, 1984; Somero, 2002; Garza and Robles, 2010). For example in the Pacific coast of North America, the thermal limit of the seastar *Pisaster* may prevent its excursion into the mid intertidal zone, where its prey (such as the mussel *Mytilus californianus*) can then survive (Fly *et al.,* 2012, Monaco *et al.,* 2015).

## Conclusions

The development of fine-scale, spatially explicit models of physical structure, temperature and ultimately physiological performance can provide critical insights into the impacts of climate change and the potential role of small-scale refugia in driving much larger-scale, geographic patterns. Specifically, the modelling framework we present shows why the relationships between these different data layers can be highly non-linear and therefore urges extreme caution when extrapolating from structural complexity (EH) and microclimate diversity. While the case study shown here has direct relevance to rocky intertidal systems, it also has applicability to other ecosystems, especially those where temperature is largely driven by patterns of solar radiation (Pincebourde *et al.,* 2007; Sears *et al.,* 2016). Moreover, conceptually these same principles apply to other biophysical processes such as water or air flow, which are comparably influenced by physical structure (Lenihan *et al.,* 2008; Denny and Gaylord, 2010; Hurd, 2015). With an increasing emphasis on the potential importance of within-site variability in selective regimes and physiological sensitivity (Dong *et al.,* 2017), quantitative methods for evaluating small-scale microclimates will continue to play a crucial role in forecasting ecological impacts of climate change and in informing conservation efforts to contend with these challenges (Rilov *et al.,* 2019).

## Supplementary Material

Supp_coz028
